# Rapid Removal of Acid Red 88 by Zeolite/Chitosan Hydrogel in Aqueous Solution

**DOI:** 10.3390/polym14050893

**Published:** 2022-02-24

**Authors:** Endar Hidayat, Hiroyuki Harada, Yoshiharu Mitoma, Seiichiro Yonemura, Hadi Imran A Halem

**Affiliations:** 1 Graduate School of Comprehensive Scientific Research, Prefectural University of Hiroshima, Shobara 727 0023, Japan; hidayatendar1@gmail.com (E.H.); mitomay@pu-hiroshima.ac.jp (Y.M.); yone@pu-hiroshima.ac.jp (S.Y.); hadiimran95@gmail.com (H.I.A.H.); 2 Faculty of Bioresources Science, Department of Life and Environmental Science, Prefectural University of Hiroshima, Shobara 727-0023, Japan

**Keywords:** acid red 88, adsorption, chitosan, dye removal, zeolite

## Abstract

In the present study, we developed a new adsorbent product with zeolite crosslinked chitosan (ZL–CH hydrogel) to remove acid red 88 (AR88) in an aqueous solution. The effects of several factors, such as the comparison of ZL–CH hydrogel and the absence of chitosan, pH, adsorbent dosage, initial AR88 concentration, contact time, and ion strength, were determined. Obtained results showed that ZL–CH hydrogel improved AR88 removal compared to the absence of chitosan, with an adsorption capacity of 332.48 mg/g in equilibrium time of 1 min, and adding ionic strength had no significant effect. However, with optimal conditions at pH 2.0, dry ZL–CH became hydrogel due to protonation of amino and hydroxyl groups through hydrogen bonds in the AR88 solution. Volume fraction and interaction force decreased with increasing porosity, leading to an increase in adsorption capacity and swelling ratio. Experimental data of the adsorption process showed the Freundlich isotherm model. The equilibrium for adsorption and swelling kinetics studies showed and fitted a pseudo-second-order model. NaOH was successful as a desorbing agent with 93.8%, and it followed the pseudo-second-order kinetics model. The recycling process indicates great potential for AR88 removal.

## 1. Introduction

Water contamination is a significant global problem caused by chemical industry effluents such as textiles, printing, food, pharmaceuticals, and paints [1]. Textile industries are one of the main contributors to water pollution, generating 10%–15% of toxic dyes [2], which hurt human health [3]. As a result, dye contamination in wastewater is a significant issue, as it prevents light from passing through the water, reducing photosynthesis, and disrupting biota growth, including fish [4]. Among these, azo dyes are widely dominant and comprise 60%–70% [5].

Azo dyes are generally identified by the presence of two aromatic groups followed by coupling the resultant diazonium salt with an electron-donating aromatic compound connected to double bond –N=N– [6]. Numerous techniques are studied for the removal of dyes in an aqueous solution, such as ion exchange [7], adsorption [8,9,10,11,12], membrane filtration [13], fungal decolorization [14], and coagulation or flocculation [15]. However, each approach has its own set of disadvantages. Of these, adsorption is a simple method that uses an adsorbent [16,17]. Many adsorbents are successful because of their ability. Recently, scientists have focused on finding an adsorbent that is nontoxic, efficient, and faster even at higher concentrations.

Zeolites are crystalline aluminosilicate minerals with a negative surface charge neutralized by exchangeable cations [18]. Furthermore, zeolites have a high surface area [19], which was explored to remove azo dyes [6]. However, zeolites are quite effective in removing azo dye from an aqueous solution [18]. Thus, we modified zeolite with crosslinked chitosan. This has various advantages, including strong biocompatibility, nontoxicity, easy chemical modifications, and it has many functional groups (methyl, amino, and hydroxyl) [20]. Chitosan is derived from chitin by deacetylation, which does not naturally occur in the environment [21]. Because its pKa is about 6.5, which leads to protonation in a neutral solution and solubility in acidic pH, it does not dissolve in alkaline conditions [22,23]. Therefore, in the postreaction of zeolite/chitosan, sodium hydroxide was added to obtain dry zeolite/chitosan hydrogel. This reaction is a new biodegradable product with great potential to remove dyes from an aqueous solution. Kolodyńska et al. [24] successfully removed methylene blue and copper using zeolite-modified chitosan. However, there is no information about the removal of azo dyes by zeolite/chitosan hydrogel. In the present study, we examined acid red 88 (AR88) in an adsorption experiment. AR88 is one of the abundant azo dyes in the effluent of the textile industry.

## 2. Materials and Methods

### 2.1. Materials

Chitosan (CH) (CAS: 9012-76-4) (deacetylation rate (after drying ≥ 80%)) was purchased from Wako Pure Chemical Industries, Ltd (Tokyo, Japan). Synthetic zeolite (ZL) was supplied from Tosoh Ltd (Shunan-shi, Japan). Acid red 88 (AR88) (CAS: 1658-56-6), sodium hydroxide (NaOH) (CAS: 1310-73-2), hydrochloric acid (HCl) (CAS: 7647-01-0), sodium chloride (NaCl) (CAS: 7647-14-5), and acetic acid (CH_3_COOH) (CAS: 64-19-7) were purchased from Kanto Chemical Co. Inc. (Tokyo, Japan). The general characteristics of AR88 are shown in Table 1.

### 2.2. Preparation of Zeolite/Chitosan (ZL–CH) Hydrogel

The modified zeolite/chitosan hydrogel was synthesized under ambient conditions. First, 1 g of chitosan (CH) was mixed with 100 mL of 1% and 3% acetic acid (AA) for 24 h on a magnetic stirrer. Then, we mixed 25 mL of dissolved CH with 0.5 g of zeolite (ZL) for 2 h on a rotary mixture (Rotator RT-50). Afterwards, we added 25 mL of 1M NaOH for 30 min. The synthesized ZL–CH hydrogel was filtered, washed to remove any residual sodium hydroxide by distilled water, and dried at 60 °C for 48 h. Lastly, the product was crushed and sieved.

### 2.3. Adsorption Experiments

In the present study, all experiments were conducted at room temperature with a magnetic stirrer with diameter particles <100 µm. The first step compares the zeolite adsorption capacity on ZL–CH hydrogel using 30 mg of zeolite/ZL–CH hydrogel with 10 mg/L in 100 mL of AR88 solution at room temperature. After being produced, the ZL–CH hydrogel was used for the next step. The effects of initial pH, different adsorbent dosages and different initial AR88 concentrations, were investigated. Because the initial AR88 (100 mg/L) was achieved in higher adsorption capacity, we used it for the next treatment. Each sample was measured three times, and the average value was taken. The amount of adsorption capacity was calculated using Equation (1).

q_e_ = {(C_o_ − C_e_)/W}V (1)

where q_e_ is adsorption capacity (mg/g), C_o_ is the initial concentration of AR88 concentration in liquid (mg/L), C_e_ is the equilibrium of acid red 88 in liquid (mg/L), W is the weight of the adsorbent (g) of the ZL–CH hydrogel, and V is the volume of AR88 (L).

### 2.4. Adsorption Kinetics

ZL–CH hydrogel was mixed with 100 mg/L (100 mL) AR88. The AR88 supernatant was collected to measure the concentration. The amount of AR88 adsorbed at the time was calculated with Equation (2).

q_t_ = {(C_o_ − C_t_)/W}V (2)

where C_t_ is the AR88 concentration at time t (min) (mg/L), C_o_ is the initial concentration of AR88 in liquid solution (mg/L), W is the weight of the adsorbent (g), and V is the volume of AR88 solution (L).

### 2.5. Desorption Studies

Practical applications always use an adsorbent with good reusability. This study produced different desorbing agents to release AR88 from ZL–CH hydrogel. First, 30 mg of ZL–CH hydrogel was loaded with 100 mL (100 mg/L) of AR88 at a fixed pH (2.0). The dye-adsorbed ZL–CH hydrogel was soaked in a 0.01 M aqueous solution of HCl, NaCl, and NaOH at a ratio of 12 mg/40 mL (S/L). Desorption amount and rate of desorption efficiency are shown in Equations (3) and (4), respectively.

q_des_ = (C_1_/W)V (3)

%desorption = (q_des_/q_e_)V (4)

where q_des_ is the desorption amount of the dye (mg/g), %desorption is the rate of desorption efficiency (%), C_1_ represents the dye concentration of desorption supernatant (mg/L), V is the volume desorption solution (L), and W is the adsorbent mass (g).

### 2.6. Swelling Studies

Swelling behavior was studied by a gravimetric method. The ZL–CH hydrogel was left to swell in AR88 solution (100 mg/L) with pH 2.0 at room temperature. The swelling was measured for each sample at a certain time after the excess surface water had been carefully removed with wet filter paper. The swelling ratio was calculated as follows:

Swelling ratio (g/g) = (W_w_ − W_d_)/W_d_ (5)

where W_w_ is the swollen weight, and W_d_ is the dry ZL–CH hydrogel sample.

### 2.7. Swelling Kinetics Studies

Dry ZL–CH hydrogel was mixed with 100 mg/L of AR88 (100 mL) solution at room temperature. Water uptake data were analyzed with the first- and second-order kinetics in Equations (6) and (7), respectively [25].

ln[S_e_/(S_e_ − S_t_)] = K_1_t (6)

t/W = 1/(K_2_S^2^e) + t/S_e_ (7)

where S_e_ is the maximal water uptake, S_t_ is the water uptake at the time (K_1_ (min^−1^)), and K_2_ (g/g min^−1^) is the specific rate constant.

### 2.8. Swelling Structure Parameters of Zeolite/Chitosan (ZL–CH) Hydrogel

The structure of the crosslinked ZL–CH hydrogel could be described by porosity and volume fraction [26,27]. The experiment was conducted in a beaker glass using 30 mg ZL–CH hydrogel mixed with 100 mg/L AR88 solution at pH 2.0, with a contact time from 0.17 to 3 min. Porosity and volume fraction were calculated as follows:

D_l_ = mass/volume (8)

V_h_ = volume − (S_2_ − S_1_ − mass/D_t_) (9)

D_h_ = mass/V_h_ (10)

Φ = {[S_wet_ − S_dry_]/S_wet_} × {D_h_/D_l_} (11)

where D_l_ is the density of AR88 (g/cm^3^), V_h_ is the volume of hydrogel (cm^3^), S_wet_ is the mass of the wet ZL–CH composite hydrogel (g), S_dry_ is the mass of the dry ZL–CH composite hydrogel, D_h_ is the density of the ZL–CH composites hydrogel (g/cm^3^), and φ is the porosity.

Interaction constant *x* (interaction force parameter) is the interaction index between hydrogel (ZL–CH hydrogel) and solvent (AR88). Force parameters were derived using Flory–Huggins theory [28]. The force parameter can express by the effective swelling balance in Equation (12).

Ø_2_ + ln (1 − Ø_2_) + *x*Ø_2_^2^ + *v_e_*V_1_(Ø_2_^1/3^ − 2Ø_2_*f*^−1^) = 0 (12)

where Ø_2_ is the volume fraction of the swelling polymer, *V*_1_ is the molar volume of the solvent, *v_e_* is the effective crosslink density, and *f*^−1^ is the average number of active elastic chains. Because the value of *v_e_* in the system was small, it could be ignored. On the basis of the literature [29], it was modified to obtain Equation (13). Lastly, the force parameter between polymer (ZL–CH hydrogel) and water molecule (AR88) is shown in Equation (14).

Ø_2_ + ln (1 – Ø_2_) + *x*Ø_2_^2^ = 0 (13)

*x* = (1/2) + (Ø_2_/3) (14)

where x is the interaction force parameter between the ZL–CH hydrogel and AR88. Volume fraction Ø_2_ could be calculated by Equation (15).

Ø_2_ = 1 − {[S_wet_ − S_dry_]/S_wet_}/{(S_dry_/D_h_) + {(S_wet_-S_dry_)/D_l_}} (15)

### 2.9. Characterization

Liquid solution AR88 was measured using a UV–vis spectrophotometer (Jasco V-530, Jasco Corporation, Tokyo, Japan) at 503 nm. The morphology of zeolite and ZL–CH composites was analyzed using scanning electron microscopy (SEM) (Hitachi TM3000, Hitachi High-Tech Co., Ltd, Tokyo, Japan). Element composition was analyzed using SEM-EDS (JIED-2300) (Shimadzu, Kyoto, Japan). The ATR-FTIR of the ZL–CH hydrogel was investigated before and after adsorption at a resolution of 1 cm^−1^ in the region of 400–4000 cm^−1^ (Thermo Scientific Nicolet iS10, Thermo Fisher Scientific Inc., Waltham, MA, USA).

## 3. Results and Discussion

### 3.1. Adsorbent Morphology 

**Figure 1:**
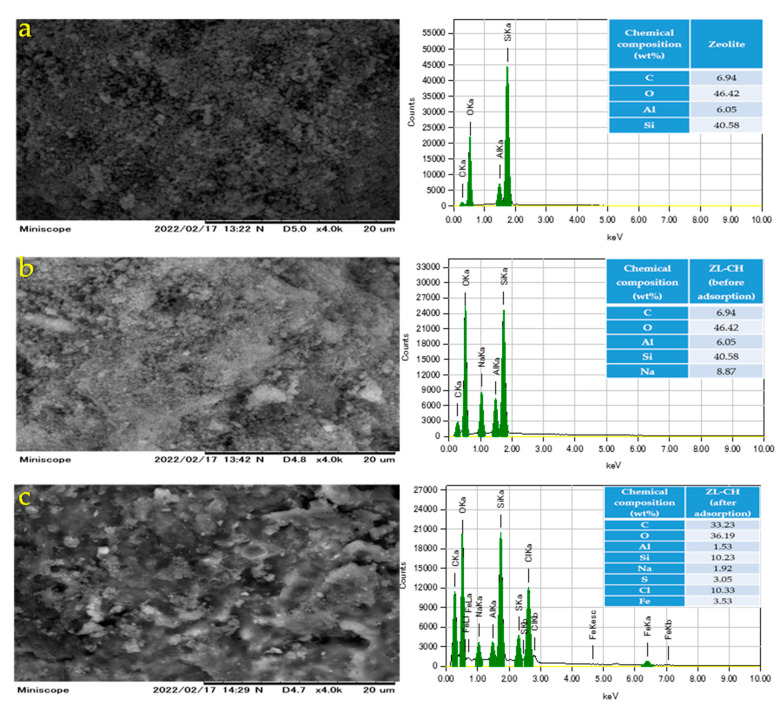
Morphology of adsorbent. SEM images (left) and EDS spectra (right). (**a**) Zeolite; (**b**) dry ZL–CH hydrogel (before adsorption); (**c**) dry ZL–CH hydrogel (after adsorption).

The photograph and EDS analysis of the raw zeolite and ZL–CH hydrogel before and after adsorption are shown in Figure 1. As seen, the zeolite surface was nonadhesive (Figure 1a). On the other hand, the dry ZL–CH hydrogel before adsorption had an interlayer and adhesive on the surface (Figure 1b). Surface changes may have been due to dissolved chitosan molecules adsorbed onto the zeolite surface. Figure 1c shows that the dry ZL–CH hydrogel surface had a rough texture after adsorption. Furthermore, EDS spectrum analysis showed that the raw zeolite Si/Al ratio (6.7/1). The sodium peak appeared in ZL–CH due to the pretreatment of the adsorbent, which consisted of sodium. Sulfur, chloride, and iron were visible after adsorption, and confirmed that the dye particles of AR88 adhered to the adsorbent surface.

### 3.2. Comparison Adsorption Capacity between ZL–CH Hydrogel and Absence of Chitosan

**Figure 2:**
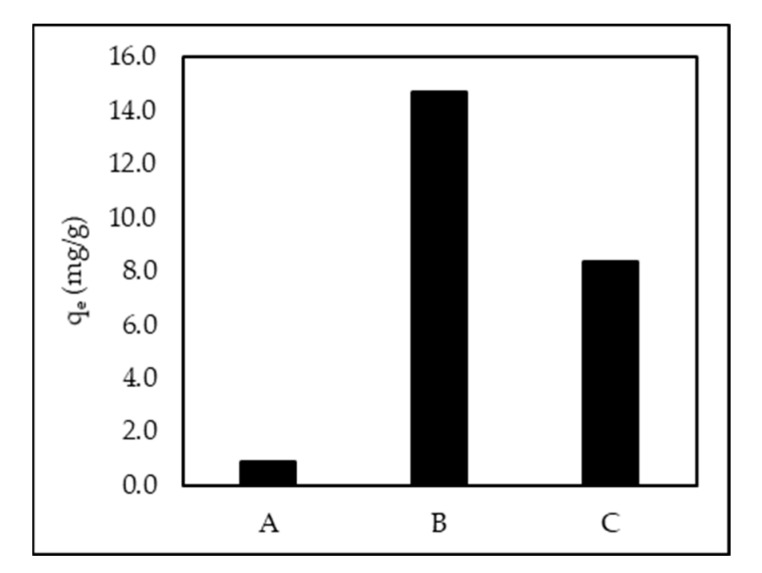
Comparison of adsorption capacity between ZL–CH hydrogel and absence of chitosan. (**A**) Original zeolite, (**B**) chitosan dissolved with 1% acetic acid (AA), (**C**) chitosan dissolved with 3% acetic acid (AA) (ZL–CH hydrogel 30 mg, 100 mL of 10 mg/L AR88, Original pH of AR88 (6.5), contact time 15 min).

Figure 2 shows a comparison of AR88 removal from the ZL–CH hydrogel and the absence of chitosan. The average adsorption capacity of ZL–CH hydrogel was greatly impacted by the absence of chitosan, which caused the molecular interactions between zeolite and chitosan to be active surfaces to capture AR88 dye particles. On the other hand, a higher concentration of acetic acid (3%) decreased adsorption capacity. This indicates that the higher concentration of acetic acid would be higher than the dissolution of chitosan, and the surface tension would be decreased [30].

### 3.3. Effect of Different Initial pH and pH Zero Point Charge (pH_zpc_)

**Figure 3:**
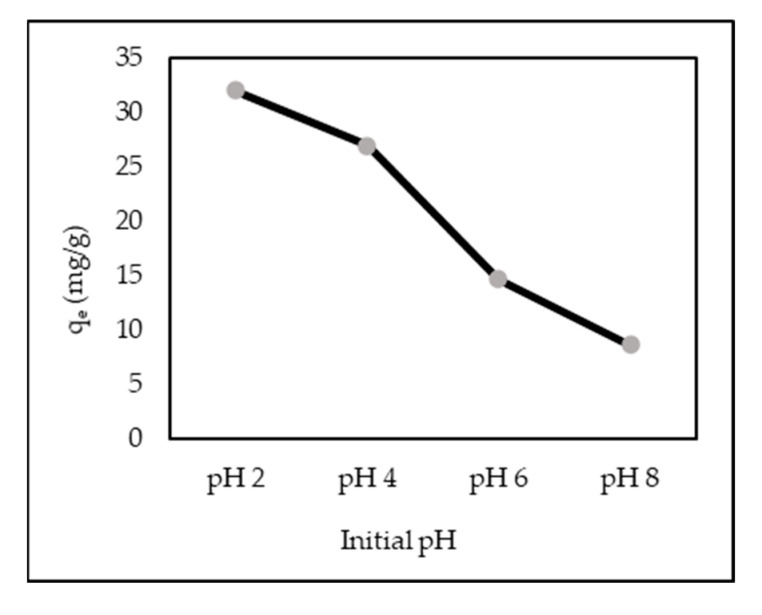
Effect of different initial pH of AR88 solution (ZL–CH hydrogel 30 mg, 100 mL of 10 mg/L AR88, contact time 15 min).

The different initial pH of AR88 in the ZL–CH hydrogel was determined in a pH range from 2.0 to 8.0 at an AR88 concentration of 10 mg/L (Figure 3). Obtained results showed that adsorption capacity decreased from 3.2 to 0.9 mg/g for pH 2.0 to 8.0, respectively. This was due to the formation of sulfonic acid groups (R–SO_3_^–^) from protonated AR88 to form R–SO_3_H under acidic conditions. However, the dye molecules dispersed, trapped inside the adsorbent, and became a (water-soluble) gel. These phenomena were due to the strong ionic interaction between the amino groups (–NH_2_) of ZL–CH hydrogel and AR88 through hydrogen bonding (H bond) [31,32,33]. However, even more so under acidic conditions, sulfonic acid groups still exhibit a pKa value below zero [34]. This agrees with [9], which reported that anionic dye adsorption was preferred under acidic conditions. However, AR88 still adsorbed on ZL–CH under alkaline conditions (pH 8). This was due to the interacting hydrogen bond between both ZL–CH hydrogel and AR88 in weak capacity. In addition, Figure S1a shows the value of pH > pH_zpc_ at an initial pH range of 2–10. This indicates that the surface area of ZL–CH was negatively charged. In comparison, the pH_zpc_ of zeolite was 5.5 (Figure S1b), which indicated that the zeolite surface was positively charged, which was not efficient to uptake dye particles in the initial pH 6.5 (Figure 2).

### 3.4. Effect of Adsorbent Dosage

**Figure 4:**
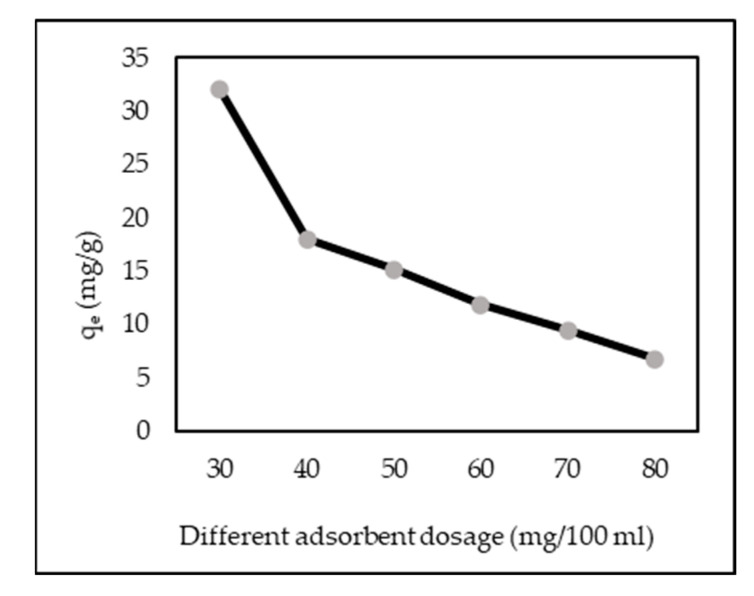
Effect of different adsorbent dosages (ZL–CH hydrogel) (pH 2.0, 100 mL of 10 mg/L AR88, contact time 15 min).

Figure 4 shows the effect of different adsorbent dosages (30–80 mg/100 mL of solution) on AR88 adsorption in an aqueous solution. By increasing the dosage, the adsorption capacity of ZL–CH hydrogel decreased from 32.0 to 6.7 mg/g. The reduction in AR88 adsorption capacity at a higher dosage of ZL–CH hydrogel may have been due to the lower accessibility of the active adsorbent sites resulting from the collection and overlap of the adsorbent particles [35]. In other words, the phenomenon that happened in the higher content of the adsorbent caused a reduction in the uptake of dye molecules into the ZL–CH hydrogel. Therefore, the remaining experiments were performed at 30 mg/100 mL of the ZL–CH hydrogel dosage.

### 3.5. Effect of the Initial AR88 Concentration

**Figure 5:**
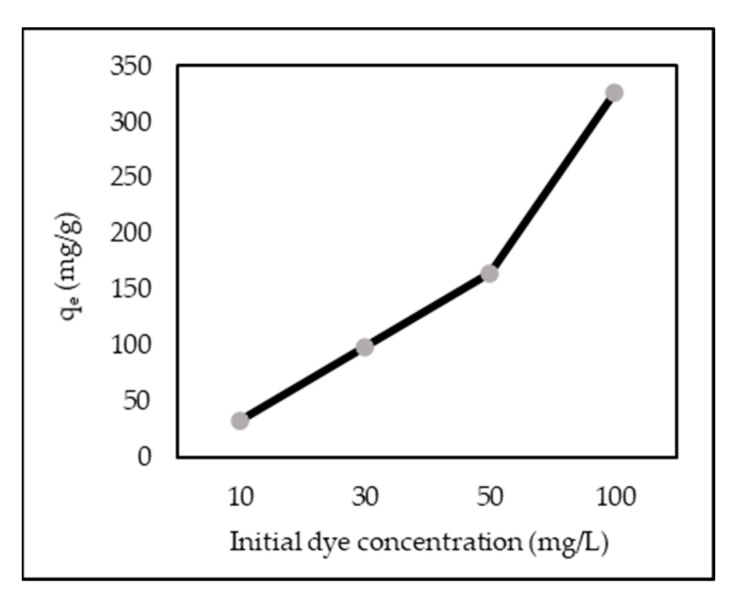
Effect of initial AR88 concentrations (ZL–CH hydrogel 30 mg, volume 100 mL, pH 2.0, contact time 15 min).

Figure 5 shows the effect of different initial concentrations of AR88 from 10 to 100 mg/L. Results show that an increase in initial AR88 concentration increased adsorption capacity from 33.2 to 326.2 mg/g.

This is attributed to the higher initial concentration due to the increased driving force between the liquid and solid phases, and the highly swollen ZL–CH hydrogel network, which provided an accessible diffusion path for AR88 dye molecules to interact with ZL–CH hydrogel chains, thus improving the uptake of the dye into the adsorbent [25,36]. While the amount of dye adsorbed increased with increasing AR88 concentration, the adsorption percentage decreased from 99.73% to 97.87%. This was due to the electrostatic repulsion between AR88 dye molecules with increasing AR88 concentration, which lead to competition between dye molecules for limited active sites in the ZL–CH hydrogel. This result agrees with the findings of [35,36,37].

### 3.6. Effect of Contact Time

**Figure 6:**
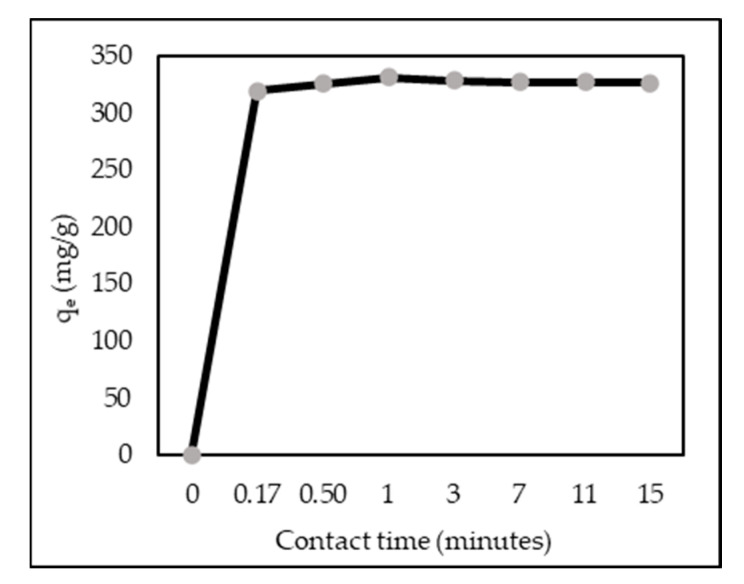
Effect of contact time (ZL–CH hydrogel 30 mg, 100 mg/L AR88 (volume = 100 mL), pH 2.0).

Figure 6 shows the equilibrium of adsorption capacity at the time. Adsorption capacity was rapid in the first 0.17 min. After 1 min, adsorption capacity reached equilibrium and gradually decreased the following time. This was due to the dissolved ZL–CH dry hydrogels, becoming gel molecules, and the electrostatic interaction with AR88 particles decreased [38].

### 3.7. FTIR and Proposed AR88 Adsorption Mechanism onto ZL–CH Hydrogel

**Figure 7:**
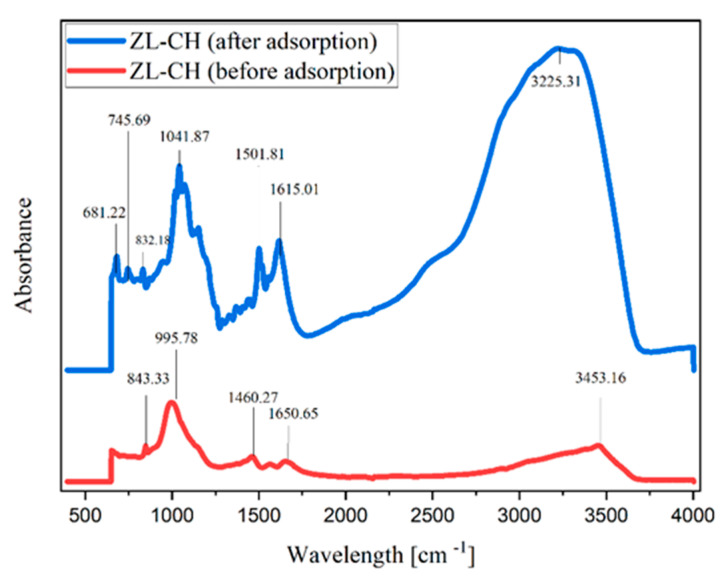
FTIR data of zeolite chitosan (ZL–CH hydrogel) before and after adsorption process.

We described the AR88 mechanism of the adsorption of AR88 on ZL–CH at pH 2.0 solution. Additional data support for the description was obtained from FTIR data of ZL–CH hydrogel before and after AR88 adsorption (Figure 7). Hydrophilic/hydrophobic functional groups existed on the surface of the ZL–CH hydrogel, hydrogen bonding, and electrostatic interactions. Before adsorption, the peak band of ZL–CH at 3453.16 cm^−1^ decreased to 3225.31 cm^−1^ after adsorption. This indicates that the hydrogen bond created between hydroxyl group AR88 and amine group of ZL–CH hydrogel at pH 2.0 was related to the stretching vibration of –OH and H_2_O [39].

**Figure 8:**
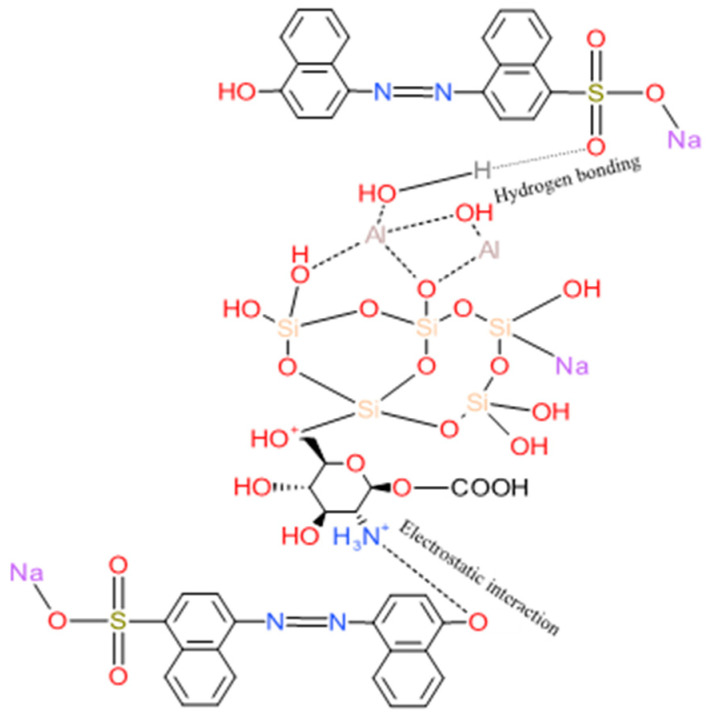
Proposed mechanisms of adsorption AR88 onto ZL–CH hydrogel.

Moreover, the decreased band peak before and after adsorption occurred at 1650.65 to 1615.01 cm^−1^, which corresponded to N–H to –OH groups; thus, the resulting electrostatic repulsion increased between ZL–CH hydrogel and AR88, leading to the color change in the solution. On the other hand, an increased band peak was found at 1460.27 to 1501.81 cm^−1^ before and after adsorption. This may have caused N–O stretching to correspond to C–H bending, resulting in OH– groups in the dye. Furthermore, new peaks after adsorption were found at 745.99 and 681.22 cm^−1^ due to the influence of aromatic bending vibrations of C–H of AR88 inserted in the adsorbent [40]. The proposed mechanism of AR88 adsorption onto ZL–CH hydrogel is shown in Figure 8.

### 3.8. Adsorption Kinetics

The kinetic adsorption process provides a valuable concept in both the action pathways and the control mechanisms of the exchange reaction. This study used the pseudo-first- and second-order models to assess the experimental data in Equations (16) and (17), respectively.

log(q_e_ − q_t_) = logq_e_ − K_1_t (16)

t/q_t_ = 1/(K_2_q^2^q_e_) + t/q_e_ (17)

where q_e_ is the amount of adsorbent (mg/g), q_t_ is the equilibrium time (mg/g), K_1_ is the rate constant of the pseudo-first-order model (min^−1^), and K_2_ is the rate constant of the pseudo-second-order model (g/mg min^−1^).

**Figure 9:**
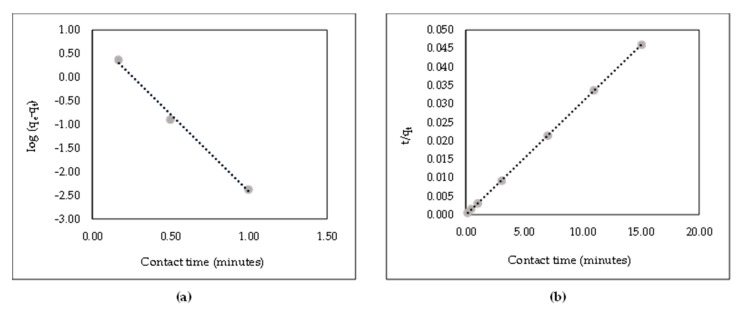
Linear kinetics plot of AR88 removal onto ZL-CH hydrogel (initial AR88 concentration = 100 mg/L, pH 2.0). (**a**) Pseudo-first-order kinetic model; (**b**) pseudo-second-order kinetic model.

Kinetic data were based on the first- and second-order log (q_e_ − q_t_) and t/q_t_, respectively. Correlation and linear kinetic plots are shown in Table 2 and Figure 9, respectively. Results showed that the experimental data of the pseudo-second-order model were in a straighter line than those of the first-order model. This suggests that the kinetic adsorption process is a second-order model.

### 3.9. Adsorption Isotherm

**Figure 10:**
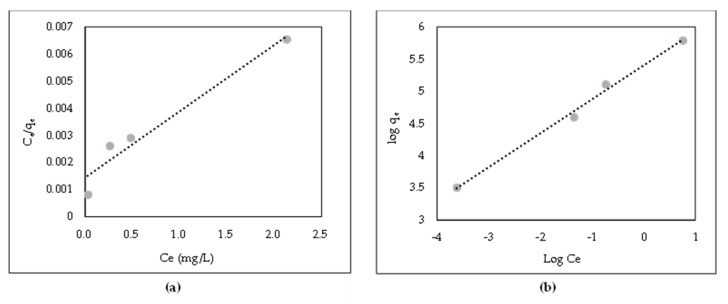
Linear isotherm plot of AR88 removal onto ZL–CH hydrogel (pH 2.0). (**a**) Langmuir isotherm model; (**b**) Freundlich isotherm model.

AR88 adsorption onto ZL–CH hydrogel and the isotherm of adsorption were as shown in Figure 10. To improve the design of AR88 adsorption processes, it is necessary to create the most appropriate linear curve relationship. In the present study, Langmuir and Freundlich isotherm models were chosen, and many scientists use them [41,42]. The equilibrium of adsorption capacity was about 332.48 mg/g.

#### 3.9.1. Langmuir Isotherm

The Langmuir adsorption isotherm model suggested that dye adsorption occurs on a uniform surface through the adsorption of a single layer. The linear Langmuir isotherm model is shown in Equation (18).

C_e_/q_e_ = (C_e_/q_max_) + 1/(K_1_q_max_) (18)

where q_e_ is the amount of the adsorbent (mg/g), q_t_ is equilibrium time (mg/g), K_1_ is the equilibrium constant of adsorption (L/mg), q_max_ is the maximal adsorption capacity of a single layer, q_e_ is the adsorbed amount on the unit mass of the adsorbent (mg/g), and C_e_ is the equilibrium concentration (mg/L).

Experimental isotherm data obtained at different initial concentrations of AR88 are shown in Figure 10a. Slope of 1/q_max,_ and interceptor of 1/K_l_*q_max_ were obtained. Furthermore, the correlation coefficient of the Langmuir constant is shown in Table 3.

The Langmuir isotherm can be depicted with a dimensionless constant separation factor, R_L_ also called the equilibrium parameter, used to describe the characteristics of the Langmuir isotherm. R_L_ was obtained from Equation (19).

R_L_ = 1/(1 + bC_o_) (19)

where C_o_ is the initial maximal concentration of AR88. The value of the separation factor (R_L_) showed the adsorption condition to be favorable (between 0 and 1), irreversible (equal to 0), and unfavorable (more than 1) on the basis of the experimental data of R_L_ in this study (1.437). Hence, the adsorption process of AR88 onto ZL–CH hydrogel was unfavorable.

#### 3.9.2. Freundlich Isotherm

The Freundlich adsorption isothermal model implied multilayer adsorption on heterogeneous surfaces. The linear Freundlich adsorption isotherm model is shown in Equation (20).

lnq_e_ = lnK_f_ + 1/n × lnC_e_ (20)

where K_f_ is related to absorbent capacity (mg/g), C_e_ and q_e_ are the same as the Langmuir isotherm adsorption model, n is the adsorption intensity that indicates AR88 and ZL–CH hydrogel adhesion. Favorability was between O and 1 [11].

Figure 10b and Table 3 show the graphs and constants of the Freundlich adsorption model, respectively. The correlation coefficient of the experimental data (R^2^ = 0.9938) was better compared to that of the Langmuir isotherm model on the basis of the calculation (n was 0.5272). This indicates that the adsorption process was the Freundlich isotherm and favorable.

### 3.10. Effect of Ion Strength

**Figure 11:**
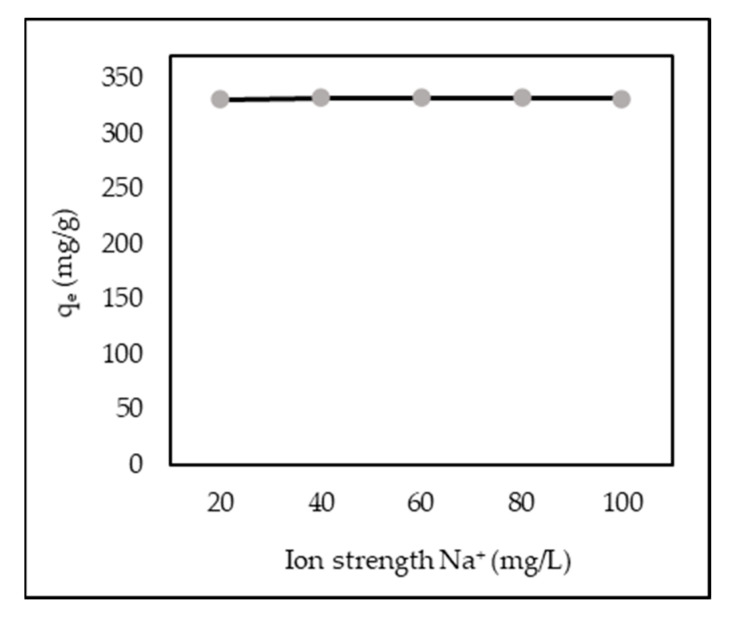
Effect of ionic strength (ZL–CH hydrogel = 30 mg, initial AR88 concentration = 100 mg/L, pH 2.0).

It is essential to investigate the effect of the solution ionic strength because these aqueous suspensions usually have different salt ion levels. Sodium chloride was used as a model salt to study the effects of electrolytes and improve the dissolution of dye molecules, thereby decreasing or increasing AR88 adsorption. AR88 adsorption capacity was established by adding various sodium chlorides. NaCl (20–100 mg/L of Na^+^ ions) into 100 mL AR88 dye solution (100 mg/L) containing 30 mg ZL–CH hydrogel with a contact time of 1 min. Figure 11 shows that the adsorption capacity of the AR88 dye solution had no impact on increasing or decreasing ionic strength. This indicated that the ZL–CH hydrogel could effectively remove AR88 from low to high ionic strength in aqueous solutions. These results agree with [35,39] for removing methylene blue, acid orange 7 by AC-ZnO, and eriochrome black T in aqueous solution.

### 3.11. Desorption Studies

**Figure 12:**
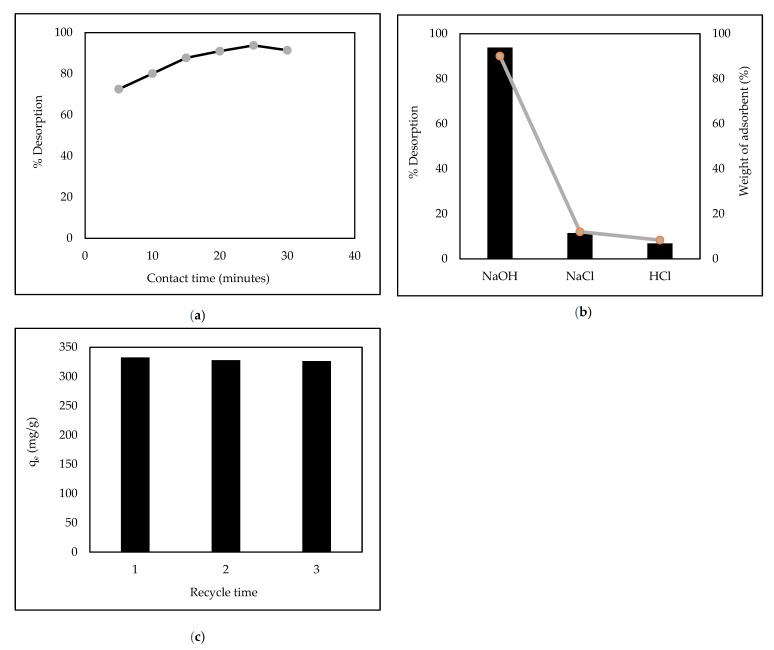
Desorption studies. (**a**) Release of AR88 in contact time by NaOH; (**b**) release of AR88 with different desorbing agents; (**c**) regeneration of AR88.

Desorption studies help in clarifying the adsorption mechanisms and recovery of adsorbents. As shown in Figure 12a, the desorption percentage of AR88 was fast after 5 min and reached equilibrium at 25 min, about 93.8% with 0.01 M NaOH solution. The, 0.01 M HCl and 0.01 NaCl were produced with the same conditions.

Obtained results showed that the desorption percentages for HCl and NaCl were about 6.8% and 11.4%, respectively (Figure 12b). This finding agrees with [43,44,45], where NaOH was very efficient in releasing azo dyes. The mechanism may have caused the detachment of hydrogen (H^+^) from the –NH^+^ to OH group of the adsorbent layer, and the hydrogen bond between ZL–CH hydrogel and the AR88 molecule could not react; then, AR88 was desorbed from AR88 loaded ZL–CH hydrogel surfaces. However, the ZL–CH hydrogel’s mass decreased to 90% after the desorption process (Figure 12b), which caused the ZL–CH hydrogel to be highly soluble in acidic conditions.

The durability of adsorbent materials is necessary, even if the adsorbent materials are regenerated several times [46]. We performed up to three recycles using the same optimal operating conditions for adsorption. As shown in Figure 12c, the adsorption capacity of ZL–CH gradually decreased as cycle time increased. Even in the third cycle, adsorption capacity was more than 326.68 mg/g, which indicated the excellent reusability of the ZL–CH hydrogel.

### 3.12. Desorption Kinetic Studies

To determine the desorption kinetics models, experimental data were fitted by first- and second-order models, as shown in Equations (21), and (22), respectively.

Log(q_des_ − q_dt_) = logq_des_ − K_1_t (21)

t/q_dt_ = 1/(K_2_q^2^q_des_) + t/q_des_ (22)

where q_des_ is the adsorbent amount (mg/g), q_dt_ is equilibrium time (mg/g). K_1_ is the rate constant of the pseudo-first-order model (min^−1^), and K_2_ is the rate constant of the pseudo-second-order model (g/mg min^−1^).

**Figure 13:**
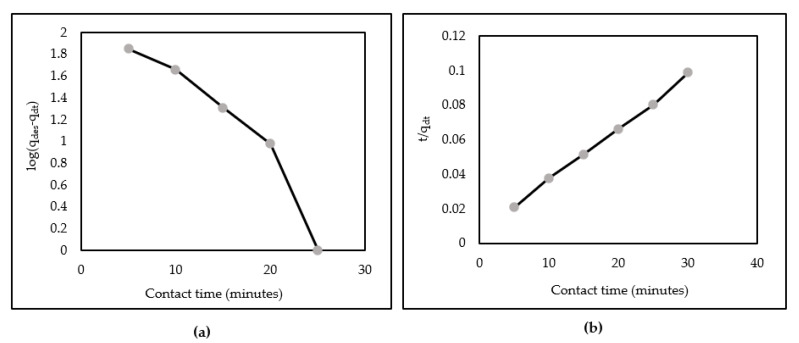
Linear desorption kinetics of AR88 from AR88 loaded ZL–CH hydrogel. (**a**) Pseudo-first-order kinetic model; (**b**) pseudo-second-order kinetic model.

The kinetic parameters are listed in Figure 13 and Table 4. The dependent coefficients (R^2^) were 0.9036 and 0.9981 for the pseudo first and second orders, respectively. Moreover, the value of the desorption experiment (q_des,_ exp) for the second order was closer to the calculation of the desorption (q_des_) than that for the first-order model. These results demonstrated that second-order desorption is well-described for kinetic models of AR88 from AR88-loaded ZL–CH hydrogel.

### 3.13. Swelling Studies

To study the effects of the dye molecules on the adsorption of the ZL–CH hydrogel, its swelling behavior was investigated at a pH 2.0 AR88 solution.

**Figure 14:**
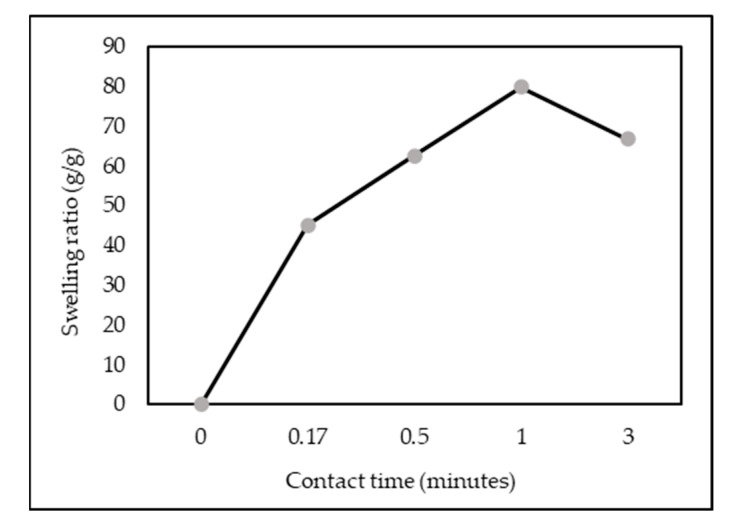
Effect of contact time of swelling studies (ZL–CH hydrogel = 30 mg, initial AR88 concentration = 100 mg/L, pH 2.0).

As shown in Figure 14, the ZL–CH hydrogel showed rapid water uptake, and the swelling ratio increased with time to an equilibrium value of 79.75 g/g at 1 min. This phenomenon was caused by the protonation of the amino (–NH^3+^) and carboxyl (–COO^–^) groups in the ZL–CH hydrogel network at low pH (2.0). Furthermore, interactions between polymer chains and silicone networks increase water permeability and resistance forces, resulting in a chain extension of molecules. As a result, the swelling ratio of ZL–CH hydrogel is increased [29].

**Figure 15:**
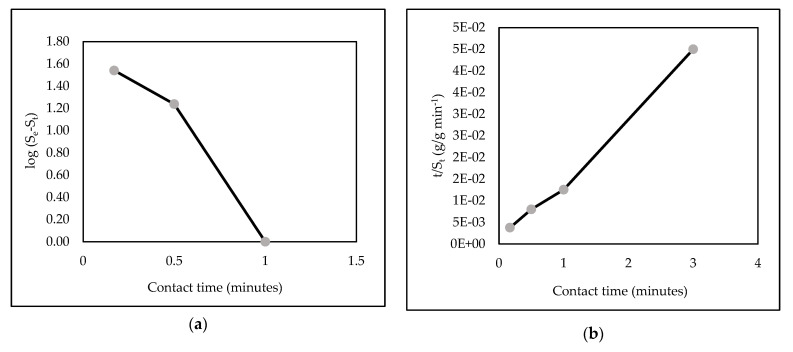
Linear swelling kinetics of AR88 from AR88 loaded ZL–CH hydrogel. (**a**) Pseudo-first-order kinetic model; (**b**) pseudo-second-order kinetic model.

The pseudo-first and second-order kinetics models of the ZL–CH hydrogel are presented in Figure 15 and Table 5. The pseudo-first-order linear coefficient (R^2^) was about 0.9526. However, significant differences between experimental (S_e_ exp) and calculated (S_e_) swelling degrees indicated that the pseudo-first-order kinetic model was unsuitable for ZL–CH hydrogel swelling processes. On the other hand, the pseudo-second-order model revealed significantly higher linearly dependent coefficients, about 0.9763, and the swelling degree (S_e_, exp) to the calculated (S_e_) was closer. These results demonstrate that the pseudo-second-order model accurately described the swelling kinetics model, corresponding to adsorption kinetics studies.

### 3.14. Analysis of Swelling Structure Parameters of ZL–CH Hydrogel

Table 6 shows the volume fraction, interaction force parameters, and porosity of the ZL–CH hydrogel.

The volume fraction and interaction force of the ZL–CH hydrogel during swelling decreased at the equilibrium time of 1 min. The reason was that ZL–CH was fully dissolved in the solution, decreased hydrophilicity, and increased intramolecular hydrogen bonding (hydrophobic groups), which led to the expansion of the molecular chain in the ZL–CH hydrogel network. In other words, protonation was higher and faster, and slowed down after 1 min. On the other hand, the porosity of the ZL–CH hydrogel increased and followed adsorption capacity and swelling degree (Figure 6 and Figure 14, respectively). This indicates that, during protonation, porosity occurred, which was the main factor for adsorbing the dye in the aqueous solution.

### 3.15. Comparison with Other Studies

The adsorption capacity of AR88 by several adsorbents is shown in Table 7. The current study indicated that ZL–CH is better than other absorbents at removing azo dye (AR88) from an aqueous solution.

## 4. Conclusions

Zeolite crosslinked chitosan (ZL–CH) hydrogel showed greatly improved AR88 removal. The adsorption capacity of AR88 decreased with increasing initial pH and adsorbent dosage, but increased with an increase in initial dye concentration. The effect of ionic strength (Na^+^) had no significant impact on the adsorption capacity of the ZL–CH hydrogel. The equilibrium of adsorption capacity was reached at 1 min with a maximal adsorption capacity of 332.48 mg/g. Adsorption capacity was fitted with the Freundlich isotherm and pseudo-second-order model. The equilibrium of swelling capacity appeared at the same time as adsorption capacity and followed a second-order kinetic model. The value of the volume fraction and interaction force parameters decreased with increasing porosity. Desorption studies showed 0.01 M NaOH to be a better desorbing agent, and it reached equilibrium at 25 min with a percentage of about 93.8%. The kinetic desorption study was fitted with a pseudo-second-order model. The recycle process was conducted for up to three cycles, which showed good capability, and that it can be used for AR88 removal.

## Figures and Tables

**Table 1:** General characteristics of AR88.

Characteristics	AR88	Chemical Structure
General name	Acid red 88	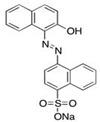
Molecular weight	400.39	
Chemical formula	C_20_H_13_N_2_NaO_4_S	
Dye type	Azo	
Nature	Anionic	
λmax (nm)	503	

**Table 2:** Correlation and constant coefficients of pseudo-first- and pseudo-second-order kinetic models for the adsorption of AR88 onto ZL-CH hydrogel.

q_e_ (exp) (mg/g)	Pseudo-First-Order Kinetic Model			Pseudo-Second-Order Kinetic Model		
	q_e_ (mg/g)	K_1_ (min^−1^)	R^2^	q_e_ (mg/g)	K_2_ (g/mg min^−1^)	R^2^
332.48	2.3546	−3.2791	0.9948	326.80	0.23	1

**Table 3:** Adsorption isotherm model of AR88 onto ZL–CH hydrogel.

q_e_ (exp) (mg/g)	Langmuir Isotherm				Freundlich Isotherm		
	q_max_ (mg/g)	K_l_ (L/mg)	R^2^	R_L_	K_f_ (g/mg min^−1^)^n^	n	R^2^
332.48	408.57	695.86	0.9497	1.437	252,688.16	0.5272	0.9938

**Table 4:** Correlation and constant coefficients of pseudo-first-order and second-order kinetic models for the desorption of AR88 from AR88 loaded ZL–CH hydrogel.

Q_des_ (exp) (mg/g)	Pseudo-First-Order Kinetic Model			Pseudo-Second-Order Kinetic Model		
	q_e_ (mg/g)	K_1_ (min^−1^)	R^2^	q_e_ (mg/g)	K_2_ (g/mg min^−1^)	R^2^
311.83	11.86	0.004	0.9036	333.33	0.0015	0.9981

**Table 5:** Correlation and constant coefficients of pseudo-first-order and -second-order kinetic models for the swelling of AR88 from AR88 loaded ZL-CH hydrogel.

S_e_ (exp) (g/g)	Pseudo-First-Order Kinetic Model			Pseudo-Second-Order Kinetic Model		
	S_e_ (g/g)	K_1_ (min^−1^)	R^2^	S_e_ (g/g)	K_2_ (g/g min^−1^)	R^2^
79.75	7.30	0.95925	0.9526	67.57	2.19	0.993

**Table 6:** Swelling structure parameters of ZL–CH hydrogel.

Contact Time (Minutes)	Volume Fraction (Ø_2_)	Porosity (Φ)	Interaction Force (*X*)
0.17	0.0110	1.957	0.5037
0.5	0.0079	1.968	0.5026
1	0.0062	1.975	0.5021
3	0.0087	1.966	0.5029

**Table 7:** Comparison of ZL–CH hydrogel with other adsorbents.

Adsorbent	q_max_ (mg/g)	Reference
Magnetic ZnFe2O4	111.1	[9]
*Lemna minor* biomass	7.8	[47]
*Sesamum indicum* seed	25	[48]
*Azolla rongpong*	78.74	[49]
Anion exchange membrane	42.01	[50]
Activated carbon	109	[51]
ZL–CH hydrogel	408.57	This study

## Data Availability

Not applicable.

## Supplementary Materials

The following supporting information can be downloaded at: https://www.mdpi.com/article/10.3390/polym14050893/s1, Figure S1. pH zero point charge (pHzpc). (a) ZL-CH hydrogel (1% acetic acid); (b) zeolite.
