# Supplementary figures and images for: Rapid Removal of Acid Red 88 by Zeolite/Chitosan Hydrogel in Aqueous Solution
Source: Polymers (Basel). 2022 Feb 24;14(5):893. doi: 10.3390/polym14050893 (PMC8912896; doi:10.3390/polym14050893)

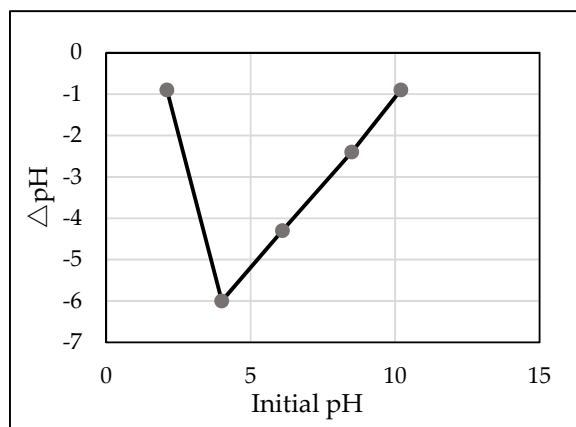

(a)

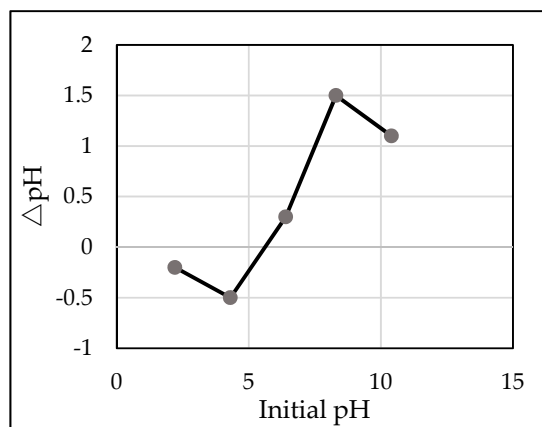

(b)

**Figure S1.** pH zero point charge ( $\text{pH}_{\text{zpc}}$ ). **(a)** ZL-CH hydrogel (1% acetic acid); **(b)** zeolite.

Supplement: Supplementary file 1 [file polymers-14-00893-s001.zip › polymers-1599487-Supplementary.pdf]
